# Assessing Urban Food Security Challenges in Gondar, Ethiopia: A Systematic Study on Household Vulnerability and Policy Implications

**DOI:** 10.1155/tswj/5867354

**Published:** 2025-03-24

**Authors:** Ishfaq Ahmad Malik, Showkat Ahmad Shah

**Affiliations:** ^1^Department of Economics, Debre Berhan University, Debre Berhan, Ethiopia; ^2^Department of Economics and Finance, University of Hail, Hail, Saudi Arabia

**Keywords:** binary logistic model, food insecurity, food security, Foster–Greer–Thorbecke

## Abstract

Food security is a paramount issue, particularly in developing nations like Ethiopia. Much of the existing research focuses on identifying the factors and constraints of households related to rural areas. However, little attention has been paid to urban householders' food security and insecurity. To this end, a study has been conducted in Gondar to evaluate urban household food security, sampling 357 homes. Results revealed that 67% of male-headed households were food secure and 33% were food insecure, while 72% of female-headed households were found food insecure and 28% were found food secure. Tenants faced higher insecurity (77%) versus homeowners (12%). The Foster–Greer–Thorbecke model highlighted 53% incidence, 14% depth, and 5.4% severity of insecurity. Tackling 15% of calorie needs could eradicate it with proactive regional governance. Binary logistic modelling identified gender, homeownership, income, marital status, remittance, and credit access as significant factors (*p* < 0.01). The study underscores the need for tailored programs focusing on income, stable pricing, housing, and low-interest loans which are crucial for addressing overcoming urban food insecurity.

## 1. Introduction

Urban poverty encompasses the economic and social challenges faced by individuals in urban areas, often characterized by insufficient access to essential needs like housing, education, healthcare, and job opportunities [1]. This form of poverty is closely linked to food insecurity, as low-income urban families often struggle to obtain affordable and nutritious food, leading to poor diets and an increased risk of malnutrition. The sustenance and well-being of humanity depend on the nourishment provided by food, furnishing essential energy for daily endeavours. It stands as a cornerstone human requirement, pivotal for human existence and advancement [2, 3]. Nonetheless, the escalating concern of food insecurity arises when there is uncertainty regarding the availability of adequate, safe, and nourishing sustenance due to economic and physical access challenges [4].

Global food security concerns stem from rising food insecurity, marked by uncertainty in accessing enough nourishing food due to economic and physical barriers [4]. Between 2021 and 2022, global undernourishment, measured by Sustainable Development Goal (SDG) Indicator 2.1.1, saw slight progress but remained significantly higher than pre-COVID-19 levels, affecting about 9.2% of the global population in 2022, up from 7.9% in 2019. An estimated 691–783 million people experienced hunger in 2022, with around 735 million being the midrange estimate, marking a rise of 122 million compared to 2019 [5].

Escalating food security concerns are fueled by rapid urban population growth, driven by urbanization and migration patterns [6]. Urban expansion depletes food resources, heightening food insecurity in cities [7]. Urbanization and annual population influxes contribute to global urban population growth rates, which were 1.84% yearly between 2015 and 2020, projected at 1.63% between 2020 and 2025, and 1.44% between 2025 and 2030 [8]. In 2016, more than half of the global population, about 54%, lived in urban areas, a figure expected to rise to 69% by 2050 [6].

The Prevalence rate of undernourishment in Africa increased from 19.4% in 2021 to 19.7% in 2022, mainly influenced by Southern and Northern Africa. Africa has seen an increase of 11 million hungry individuals since 2021 and over 57 million since the onset of the pandemic [9]. A significantly higher percentage of Africa's population of nearly 20% experiences hunger, compared to 6.5% in Latin America, 8.5% in Asia, 7.0% in Oceania, and 6.5% in the Caribbean [10]. This problem is underscored by studies emphasizing on food security within Africa's rural impoverished communities, while the urban poor, who encounter comparable vulnerabilities, receive inadequate attention [11].

Ethiopia's national poverty rate declined from 30% in 2011 to 24% in 2016. In urban areas, poverty dropped significantly from 26% in 2011 to 15% in 2016, indicating a faster pace of poverty reduction compared to rural regions. In rural areas, poverty decreased from 30% in 2011 to 26% in 2016 [12]. Food insecurity has further strained Ethiopia's already fragile economy, particularly as migration from rural to urban areas has increased, leading to higher unemployment rates in many cities. Ethiopia's urban population growth rate was 4.2% in 2022 and with this pace, it is projected that the urban population will surpass 50 million by 2025 [13]. While Ethiopia's total population in 2022 was 123,379,924, with 23% residing in metropolitan areas, urbanization has emerged as a relatively recent development in Ethiopia, and its annual expansion rate of 4.6% outpaces the continent's average of 4%. Existing urban centers are expanding, and new ones are emerging [14].

The rapid urbanization of Ethiopian society has triggered food insecurity in major cities across the Amhara Region and nationwide, each presenting its unique set of challenges. Urban food insecurity carries distinct implications, underscoring the importance of this study in investigating the state of and factors influencing food security to enrich our comprehension of the evolving large cities in the Amhara Region. It is noteworthy that Ethiopia initiated a food security policy and food security program in 1996 and 1998, respectively [15]. Despite these endeavours, food insecurity and undernutrition persist as pressing societal concerns. Consequently, there is a considerable lack of understanding regarding the subject within the examined area. Numerous studies have attempted predominantly concentrated on rural areas, as exemplified by Aragie and Genanu [16] and Yehuala et al. [17], among others. However, these partial assessments fall short of capturing the ground-level realities and conceal Ethiopia's authentic urban food security challenges. Furthermore, these inquiries fail to explore the reasons behind household food insecurity, particularly in urban areas.

Existing literature (such as [18, 19]) predominantly relies on metrics such as caloric intake, the Household Food Insecurity Access Scale (HFIAS), the Household Hunger Scale (HHSc), or the Household Food Consumption Score (FCS) in isolation, limiting the depth and specificity of the understanding of this issue in cities. Although some other studies carried out in Ethiopia have primarily focused on areas such as access and food availability [20], some other studies have employed either the 7-day or 24-h recall method to evaluate the food aspect [21], frequently dividing it into two separate aspects. In a comprehensive examination of household food security in various regions of Ethiopia, diverse research studies present a concerning narrative. For example, Birhane et al. [22] focused on Addis Ababa, employing a three-stage sampling technique among 550 households, discovering that 75% were food insecure, with 25% experiencing hunger. Tulem and Hordafa's [23] investigation in Waliso Town reported a high prevalence of household food insecurity at 63.4%, citing family size and the employment status of the household head as significant contributing factors. Tigistu and Hegena [24] delved into Southern Ethiopia, finding that 74.17% of the households surveyed experienced food insecurity. The logit model highlighted age, educational level, sex of the household head (HHS), access to remittance, and family size as influential factors of food security status. Dinku et al. [25] adopted a mixed study design, surveying 506 households that were selected randomly in Kombolcha and Dessie. Utilizing the HFIAS, they uncovered that 33.1% of respondents faced food insecurity, with 9–6 individuals per 100 enduring hungers during the day and night, respectively. Similarly, a community-based cross-sectional study involved 377 households, revealing a 32.4% food insecurity rate, of which 10.3%, 18.8%, and 3.2% experienced mild, moderate, and severe food insecurity, respectively, Demie and Gessese [26]. Collectively, these studies underscore the urgency for targeted interventions and policy measures to address the multifaceted challenges surrounding food security in Ethiopia.

To fill up the gap in existing literature, our study employs the Foster–Greer–Thorbecke (FGT) index to comprehensively evaluate the incidence, depth, and severity of food insecurity and identify the variables influencing households' food insecurity in the urban centre of Ethiopia, that is, Gondar. The central aim of this research is to explore the unique hurdles and potential pathways toward attaining food security in an urban environment. The expected findings of this study are poised to identify precise barriers and promising avenues that can serve as invaluable guidance for policymakers, implementers, and various stakeholders in urban centre. This insight will empower them to craft and execute tailored initiatives to fortify and promote food security in urban settings. Therefore, our study provides valuable insights and contribution to the existing literature.

The remainder of the paper is structured as follows: After conceptual framework, the subsequent section outlines the methodology, followed by Section 3 which delves into the results and discussions. The final section covers conclusions and policy implications.

### 1.1. Conceptual Framework

**Figure 1:**
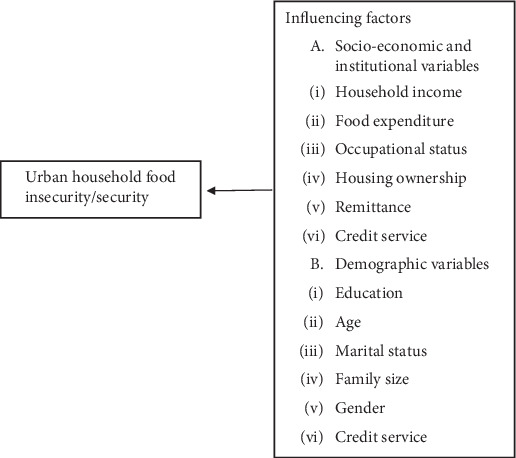
Conceptual framework. Source: made by authors (adopted from [27]).

Figure 1 illustrates the correlation between demographic, institutional, and socioeconomic factors in explaining urban food insecurity/security.

## 2. Material and Methods

### 2.1. Case Study Profile

**Figure 2:**
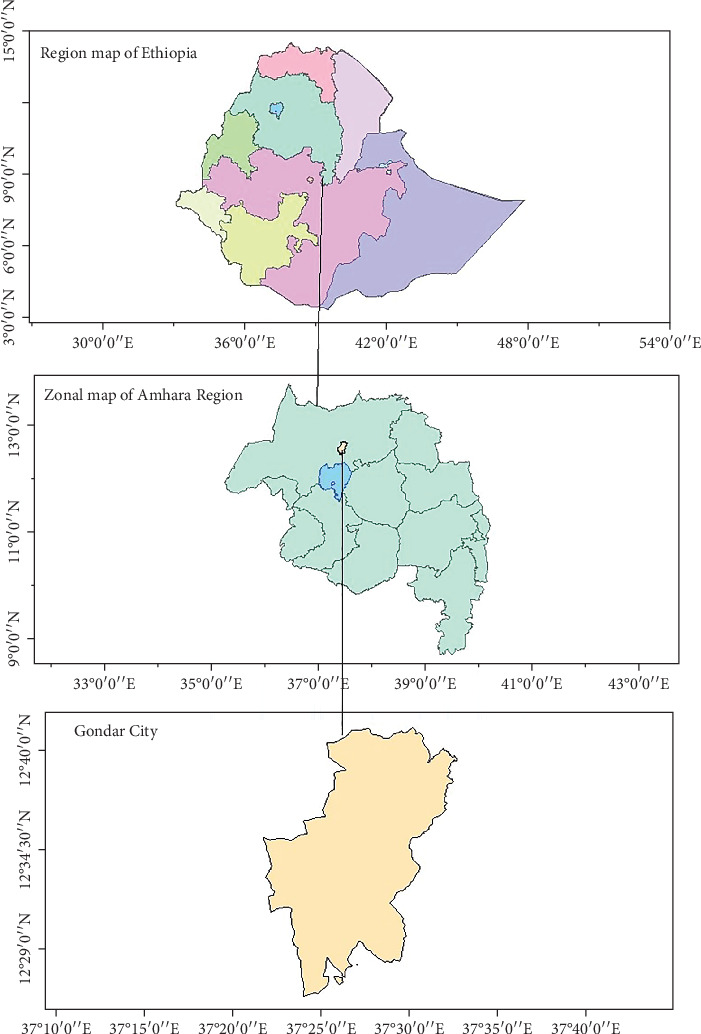
Location map of Gondar. Source: developed by author in ArcGIS tools.

Gondar is in the northwestern part of Ethiopia with the Amhara National Regional State. Gondar's precise coordinates are 12°36⁣′ N and 37°28⁣′ E. The city is situated approximately 727 km from the capital of Ethiopia (Addis Ababa). Gondar comprises six administrative subcities, namely, Jantekel, Arada, Zobel, Fasil, Maraki, and Azazo-Tsuda. According to data from the Central Statistical Authority [28], the Gondar City's recorded population is 452,188. Gondar plays a pivotal role as a magnet for rural–urban migrants and serves as a significant hub for tourism and business activities in the northwestern region of Ethiopia.  Figure 2 represents a location map of Gondar (Ethiopia).

### 2.2. Research Design

This study adopted a mixed-methods research design, integrating both quantitative and qualitative approaches. The rationale for this choice stems from the recognition that a comprehensive analysis requires the strengths of both methods; each approach may prove inadequate in capturing the complexities of a condition [29]. Given the complex and multidimensional nature of food security studies, employing both quantitative and qualitative research methods concurrently was deemed essential.

### 2.3. Sample Size and Technique

Gondar, a significant city in the Amhara Region, was intentionally chosen due to its embodiment of the characteristics typical of a large town in the region. The urban food security program and poverty reduction efforts heavily rely on the city's pivotal role in the region. Gondar consists of 22 kebeles, and for the present study, three kebeles, namely, Adebabay Evesus, Abajalie, and Azezo Dimaza, have been randomly selected to represent the entire area. The total number of registered household heads in all the sampled kebele was found to be 59,512; the sample distribution in each kebele is shown in Table 1. Sample from each kebele was chosen proportionally to the number of household heads in the kebele. Using Kothari's [30] sample determination formula, a sample of 382 respondents was calculated for the present study, as expressed below. To account for potential nonresponses or missing questionnaire, an additional sample of 5% was added. Consequently, the final appropriate sample size was determined to be 401. However, 21 respondents did not return the questionnaire, and 23 others provided unclear information. Therefore, in this study, we analyzed only 357 samples.

 $$\begin{array}{c} n=\frac{z^{2}p∙q∙N}{e^{2}\left(N-1\right)+z^{2}∙p∙q} \end{array}$$

where *n* is the sample size, *N* is the population size, *z*is the standard variation for confidence level of 95%, *p* = sample proportion = 50% (0.50), 1 − *p* = *q* [31], and *e* is the estimate which should be within 5% of true value.

### 2.4. Data Collection and Sources

Both primary and secondary data were used in this study. We employed a structured questionnaire incorporating both open- and closed-ended questions to gather demographic, socioeconomic, food security, and insecurity determinants and other institutional questions on household heads from the sampled area. The questionnaire was developed based on an extensive literature survey of concern issues and was pretested to some selected respondents to check the validity of the questionnaire and get valuable feedback to enhance the questions of the survey for the robustness of results.

Along with the household survey, supplement information was collected from key informants and focus group discussions. The adoption of these methods was driven by their effectiveness in minimizing the likelihood of nonresponses and providing the researcher with in-depth information. Four key informants from sampled kebele were chosen such as a food security expert, block-level administrator, a social activist, and a development agent to provide detailed information about the study area. They were selected based on concern, experience, and knowledge about the subject matter. Besides, some focus group discussions were also taken before the household survey. These discussions include participants that pertained to different socioeconomic backgrounds, old age, young people, and gender (male and female)- headed households. They enlightened us about the region's several issues such as food prices and supply, income sources of households, sources and access to credit services (HHCRs), and remittances. These methods were chosen to enhance not only the depth of information and minimize the potential for nonresponses but will also supplement the findings from the field survey. Besides, secondary data collection methods encompassed the utilization of accessible documents and reports from various international and national organizations and local government offices.

### 2.5. Methods of Data Analysis

In this study, descriptive statistics, a food security index, and a binary logistic model were employed to analyze the data. The descriptive statistics, including mean, frequency, and percentage, were analyzed for demographic, socioeconomic, and institutional variables of households in the study area. Inferential tests, such as the chi-square test, were used to determine the statistical significance of these variables.

Food security was evaluated by measuring dietary energy consumption at a benchmark of 2100 kcal per capita per day. This benchmark is based on the Ethiopian government's recommended weighted average food requirement per adult per day [32, 33]. Households consuming more than 2100 kcal are considered food secure, while those consuming less are deemed food insecure.

To assess food security, food quantities consumed were converted into grams, and their calorie content was determined using the 1998 food consumption table from the Ethiopian Health and Nutritional Research Institute. These estimates were adjusted to adult equivalent values, resulting in the daily food calorie intake per adult equivalent. Data on the types and quantities of food consumed households by seven consecutive days were collected using the weighted average method. This data was then converted to kilocalories, divided by the household size measured in adult equivalents and the number of days. Thus, the household's daily food calorie intake per adult equivalent (HFCI) is calculated accordingly [34, 35].

 $$\begin{array}{c} \text{HFCi}=\frac{\text{total calorie consumed by household}}{\text{household size in adult equivalent}*7} \end{array}$$

Furthermore, the study utilized the FGT, a food security index to calculate the incidence, depth, and severity of food insecurity. This index is a predominant metric employed in food security analysis, widely recognized for its effectiveness. This index, initially proposed by Foster et al. in 1984, has exhibited several desirable attributes that have been further refined for food insecurity assessments in recent years [36]. In this study, the FGT is employed to calculate the incidence, depth, and severity of food insecurity within households. The food insecurity incidence indicates the percentage of households living below the food insecurity threshold. The food insecurity depth index measures the severity of food insecurity by considering the distance of individuals from the food insecurity threshold [37, 38]. The food insecurity severity index indicates the severity of food insecurity experienced by households. As the FGT index approaches 1, the level of food insecurity increases [39, 40]. The specification of the FGT food security index is as follows:

 $$\begin{array}{c} {\text{FGT}}_{\propto }=\frac{1}{N}\overset{H}{\underset{i=1}{\sum }}{\frac{\left(Z-y_{i}\right)}{Z}}^{\propto } \end{array}$$

where *N* is the number of sampled households, *H* is the number of food-insecure households, *Z* is the cutoff between food security and food insecurity, *y*_*i*_ is the per capita food calorie intake of the *i*th household, and ∝ is the weight assigned to the severity of food insecurity.

When ∝ = 0, the FGT index represents the incidence of food insecurity. At ∝ = 1, it assesses the depth of food insecurity, indicating the proportion of the food security threshold that food-insecure households would need to reach to alleviate food insecurity. With ∝ = 2, the FGT index measures the severity of food insecurity by quantifying the distance between food-insecure households and the food security threshold. After quantifying and computing the extent of food insecurity, statistical analyses were conducted to assess significant differences among the independent variables.

Moreover, following the studies such as Feleke and Bogale [41], Aragie and Genanu [16], and Mota et al. [42], a binary logit model was found appropriate when dealing with binary dependent variables, like in our case, households classified either as food secure (coded as 1) or food insecure (coded as 0). The analysis employs a logit model, considering crucial assumptions such as concerns regarding nonlinearity and multicollinearity and ensuring an adequately large sample size.

The logit regression model was formulated with the following functional expression:

(1) $$\begin{array}{c} \pi_{\left(x\right)}=E\left(Y=\frac{1}{X}\right)=\frac{1}{1+e^{-\left(\beta_{0}+{\beta x}_{t}\right)}} \end{array}$$

For ease of exposition, we write (1) as

(2) $$\begin{array}{c} \pi_{\left(x\right)}=\frac{1}{1+e^{-z_{i}}} \end{array}$$

where *π*_(*x*)_ is the probability of being food secure ranging from 0 to 1 and −*z*_*i*_ is a function of explanatory variables (*X*_*i*_) which is expressed as

(3) $$\begin{array}{c} z_{i}=\beta_{0}+\beta_{1}X_{1}+\beta_{2}X_{2}+\beta_{3}X_{3}+\beta_{4}X_{4}+\beta_{5}X_{5}+\beta_{6}X_{6}+\beta_{7}X_{7}+\beta_{8}X_{8}+\beta_{9}X_{9}+\beta_{10}X_{10}+\beta_{11}X_{11}+U_{i} \end{array}$$

The parameters of the model (*β*_0_ + *β*_*i*_) have been estimated using the maximum likelihood (ML) method [43, 44].

*β*_*i*_ represents coefficients; *X*_1_, HHS; *X*_2_, household marital status (HHMS); *X*_3_, household remittance (HHR); *X*_4_, HHCR; *X*_5_, HHOWN; *X*_6_, household food expenditure (HHFE); *X*_7_, household income (HHI); *X*_8_, age of the household head (HHA); *X*_9_, household family size (HHFMZ); *X*_10_, household education (HHEdu); and *X*_11_, household employment status (HHEMS). The description and expected signs of the explanatory variables are presented in Table 2.

## 3. Results and Discussion

### 3.1. Demographic Variables Versus Household Food Security Status

The study results reveal a distinct contrast in food security levels between households led by males and those led by females. As shown in Table 3, 67% of male-headed households were food secure, while 33% were food insecure. In contrast, 72% of female-headed households were food insecure, with only 28% achieving food security. This observed difference highlights a significant pattern, suggesting that female-headed households are more susceptible to heightened food insecurity compared to those led by males. This pattern aligns with the study conducted by Ashagidigbi et al. [45] who specifically identified the female gender as the predominant food insecure and disempowered group in Nigeria. Moreover, Agidew and Singh's [46] study focussed on Ethiopia, indicated that, on average, households led by men tended to experience higher levels of food security compared to those led by women.

The HHMS varies dynamically. Among 193 married respondents, 69% reported food security, while 31% were food insecure (Table 3). In contrast, of 163 single-headed households, 80% faced food insecurity, with only 20% achieving security. This correlation suggests that joint financial contribution in married households reduces expenses compared to separate living, promoting stability in food security. This aligns with Bogale et al. [47], who found enhanced food security in married households due to better access to productive assets by male partners. Tadesse et al. [48] also support this, indicating that coupled households tend to pool resources, boosting income compared to singles. These findings underscore the complex interplay between HHMS, economics, and food security.

HHFMZ significantly influences food security. The hypothesis suggests a negative link between household size and food security due to more mouths to feed with limited resources. Data shows smaller households (1–3 members) with 47% food secure, while larger ones struggle more, with 53% facing insecurity. Similarly, 4–6-member households had 42% food security, contrasting with 58% insecurity. Those exceeding seven members had only 37% food security. These findings echo Mengistu and Kassie [49], highlighting that as household size grows, achieving food security becomes harder. This underscores the pivotal role of household size in shaping food security outcomes.

The research identified a favourable correlation between HHA and the level of food security. Among the 79 respondents falling in the age group of 20–40, 42% achieved food security, while 58% experienced food insecurity. In the age group of 41–60, encompassing 264 respondents, 55% reported food security, with 45% facing food insecurity (Table 3). Finally, the age group of 61–80 predominantly grappled with food insecurity. This observed relationship can be attributed to the notion that the productivity of older household heads tends to increase with age. As household heads age, their accumulated experience in various activities contributes to enhanced productivity. This outcome aligns with the findings of Gebre et al. [50] supporting the idea that older household heads tend to exhibit higher food security, possibly due to increased expertise and productivity over time.

The study reveals a vital link between HHEdu levels and food security. Among households with illiterate heads (33% of the total), 45% are food secure, while 55% face insecurity. Conversely, 67% of heads possess some education, showing better food security prospects. Research by Smith and Haddad [51] underscores education's role in enhancing resource utilization for improved food security. Similarly, Ruel and Alderman [52] found maternal education positively impacting child nutrition, highlighting education's broader influence on food security. Studies by Barrett and Heisey [53] and Rose et al. [54] emphasized that education's role in income generation is crucial for ensuring adequate food supply at the household level.

### 3.2. Socioeconomic and Institutional Variables

Among respondents spending 1500–2500 birr monthly on food, a staggering 88% faced insecurity, with only 12% secure. Conversely, of 111 spending 2501–3500 birr, 84% experienced insecurity, with the remainder secure. Notably, as expenditure rises (3501–4500, 4501–5500, and above 5500 birr), food security percentages also increase, aligning with Rose et al. [54] and Amao et al. [55]. Higher expenditure implies more income sources and reduced insecurity. However, escalating food prices can strain urban households, dedicating most income to food and signalling poverty.

Income plays a significant role in determining households' food access. Survey data indicates a direct relationship between monthly income and food security, with higher income levels closely associated with improved food security. Among households earning less than 2500 birr monthly, 88% face insecurity, while those earning over 4500 birr are all secure. This aligns with expectations as higher income boosts purchasing power, aiding in navigating price dynamics.  Table 4 data shows that higher daily income per adult correlates with lower food insecurity. Silvestri et al.'s [56] and Etea et al.'s [57] findings support income's dominant role in food security, outweighing other factors.

Remittance recipients refer to households that receive income from overseas sources. According to our study, 35% of households receive remittances, with 86% of them being food secure and 14% experiencing food insecurity. Conversely, 65% of households do not receive remittances, and among them, 35% are food secure while the remaining households face food insecurity. Similarly, the study of Szabo et al. [58] suggested that remittances have a significant positive effect on food security.

Occupational status significantly impacts income and subsequently food spending. Approximately 50% of participants were paid workers, with 52% achieving food security due to higher income. The remaining 50% engaged in diverse occupations like daily labor and small-scale enterprises. Among them, 42% were food secure, showcasing financial stability. However, 58% faced insecurity, highlighting economic challenges in alternative occupations. Bahiru et al. [59] and Mengistu and Kassie [49] similarly found such trends.

Households are categorized into two groups: tenants and homeowners. As depicted in Table 4, tenants constitute the majority, with 65%. Among them, a significant 77% were food insecure, while only 23% managed to achieve food security. In contrast, homeowners represent 35% of all sampled households, with the majority (88%) in the food-secure category and only 12% facing food insecurity. This data underscores the critical role of HHOWN in urban areas in ensuring household food security. The study of Dinku et al. [25] found the same results.

HHCR refers to providing loans or credit by financial institutions to support financial needs or investment [60]. Our results reveal that 60% of households lack access to HHCRs, and among them, a substantial 70% experienced food insecurity, while the rest maintained food security. In contrast, households with access to credit represent 40% of the sample. Among these, 63% reported food security, with 37% confronting food insecurity. Consequently, households with credit access are more prone to invest in diverse activities and ultimately achieve food security [61]. Hence, it is hypothesized that a family with access to credit is more likely to attain food security. Therefore, households having credit access are more likely to achieve food security.

### 3.3. Food Security Status by FGT

Table 5 presents measures related to food insecurity, each with their corresponding values and percentages. The incidence of food insecurity (*P*_0_) measures the proportion of the population experiencing food insecurity, revealing that 47% of the households meet their minimum substance requirement, while the remaining 53% of sampled households (189) fall below the daily intake of 2100 kcal per day. Additionally, the depth of food insecurity (*P*_1_) measures the food insecurity gap experienced by households, indicating an average severity of 14%. This means that each food-insecure household required an additional 14% of their daily calorie requirements to reach the threshold level of 2100 kcal, thereby eliminating food insecurity. Furthermore, the severity of food insecurity (*P*_2_), which is the average of squared gaps in food consumption from the food security threshold, quantifies the severity of food insecurity in households with a value of 5.4%.

The results of this study, along with findings from existing studies, demonstrate a mix of outcomes, with some studies showing proximity while others exhibit significant disparities. For instance, Mekonen et al. [62] investigation on urban Amhara revealed values of *P*_0_ at 54%, *P*_1_ at 15%, and *P*_2_ at 5.6%. In contrast, Welderufael's study in [63] found *P*_0_ to be 34%, *P*_1_ to be 6%, and *P*_2_ to be 2%. Another study by Aragie and Genanu in 2017, which focused on food security levels and determinants in the Wollo Zone, reported that 58% of households were food secure, with the remaining 42% being food insecure. Additionally, their study indicated *P*_1_ at 14% and *P*_2_ at 7.15%. Similarly, Feleke and Bogale's study in 2010 conducted in Dire Dawa observed *P*_0_ at 43%, *P*_1_ at 13%, and *P*_2_ at 5.9%. These diverse findings underscore the variability in food security levels across different regions and highlight the importance of context-specific interventions and policies.

### 3.4. Determinants of Household Food Security

A binary logistic regression model was utilized to ascertain the factors influencing the food security of households. Eight out of the 11 predictor variables showed significant associations at a 1% (*p* < 0.001) probability level as shown in Table 6. The coefficients of the model yielded a 2.41 value of chi-square, corresponding to 1 degree of freedom, indicating strong statistical significance at *p* < 0.01. This suggests that predictor variables have a collectively substantial impact on the status of food security. The value of (0.092) Hosmer and Lemeshow's test also indicates that a model is a well-fitted model for the study. Moreover, the model exhibited a strong predictive capability, correctly classifying 92% of the 357 sample households included in the analysis. We found no significant violations among independent variables after practicing the multicollinearity test. The model's explanatory variables, evaluated through pseudo-*R*^2^, revealed that the combined independent variables accounted for approximately 77.8% variance in the model.

HHS is a noteworthy predictor (*p* = 0.003) for achieving food security. An increase of one unit in male-headed households raises the likelihood by 14.56 times, with a 95% confidence interval (CI). This echoes Aragie and Genanu's [16] study, showing higher food insecurity among female-headed households. Conversely, male-headed households, when other factors are constant, are more prone to food insecurity. This could stem from female heads' increased family focus, enhancing caloric availability due to gender-based spending priorities [41].

HHMS stands out as another positive and significant predictor, with a *p* value of 0.002, for achieving food security. The odds ratio (OR) indicates that with an increase of one unit in married households, the odds of achieving food security rise by a factor of 17.45 (indicating higher odds), with a 95% CI ranging from 2.98 to 98.67. This result is consistent with the conclusions drawn by Feleke and Bogale [41], indicating that households with married couples significantly contribute to improving food security. This is attributed to the economic efficiency achieved through joint consumption decisions and the consolidation of resources. Consequently, married households effectively minimize individual expenditures that would otherwise be incurred independently.

Household remittance received (HHRR) significantly influences food security (*p* = 0.000), with each unit increase substantially boosting the odds by 75.23. The 95% CI spans from 15.43 to 365.87, affirming remittance's robust impact on food security. This positive association indicates that higher remittance inflows enhance the likelihood of achieving and maintaining food security. The findings of this study are consistent with Abadi et al. [64], who found that remittance-receiving households in Tigray, Ethiopia, experienced improved food security, reflected in lower Coping Strategy Index (CSI) and HFIAS scores. Similarly, the study of Smith and Maria [65] reported that remittances more effectively reduce food insecurity in lower-income countries than in middle-income ones, highlighting the influence of economic development level.

Access to HHCRs significantly predicts food security (*p* = 0.000). Each unit increase boosts the likelihood by 14.34 times, with a 95% CI of 4.45 to 70.67. This highlights HHCRs' crucial role in enhancing food security by providing opportunities for alternative income generation, bolstering financial capacity, and reducing vulnerability to insecurity. The findings align with Feleke and Bogale [41], illustrating a negative association between this variable and food insecurity.

HHOWN shows a positive and significant predictor (*p* = 0.000) of the probability of attaining food security. The OR indicates that an increase of one unit in HHOWN and the odds of achieving food security increase by a factor of 38.67 (indicating higher odds), with a 95% CI ranging from 18.78 to 198.56. The positive coefficient signifies that increased HHOWN influences the balance. HHOWN can lead to a decrease in rental expenses, which in turn impacts expenditure. Consequently, HHOWN contributes to more stable income and enhanced capacity to afford food, ultimately increasing household food security. Fafard St-Germain and Tarasuk [66] found that food insecurity prevalence was highest among renters compared homeowners.

HHI is another significant and positive predictor (*p* = 0.003) of the likelihood of achieving food security. The OR indicates that with each unit increase in HHI, the likelihood of achieving food security significantly rises by a factor of 198.45 (signifying considerably higher odds), with a 95% CI spanning from 47.34 to 2950.54. This demonstrates the critical role of income in determining a household's access to food. As monthly income increases, food security tends to improve, mainly due to enhanced purchasing power and increased access to food resources. Etea et al. [57] found that HHI levels in Ambo District, Ethiopia, were low, with the majority of households experiencing food insecurity. Through binary logistic analysis, they determined that income significantly and positively contributes to food security in the district.

HHFE of households emerges as a pivotal factor influencing food security, as underscored by its positive and statistically significant association in this study (*p* = 0.000). The OR further illuminates this relationship, revealing that for every incremental unit increase in HHFE, the odds of achieving food security amplify significantly by a factor of 120.67. This substantial increase in odds is encapsulated within the 95% CI, which spans from 13.23 to 1243.05. Essentially, this signifies that households dedicating a higher proportion of their income to food expenses are more likely to experience food security, establishing a direct and positive correlation. Conversely, lower HHFE corresponds to a diminished likelihood of achieving food security. The results of the study are consistent with those of Abafita and Kim [67], who found a strong and positive relationship between per capita consumption expenditure and household food security. Similarly, Abegaz [68] demonstrated that HHFE plays a significant role in influencing food security in Ethiopia, with increased income enabling households to spend more on food, thereby improving their access to and security of food.

The age of the household head (HHA) significantly and positively impacts their food security status. HHA emerged as a noteworthy and positive predictor (*p* = 0.006) of the likelihood of achieving food security. The OR indicates that for each unit increase in the age of the household head, the odds of attaining food security have amplified by a factor of 5.798 (signifying a heightened likelihood), with a 95% CI spanning from 3.143 to 25.76. Holding other variables constant, an increase in the age of the household head results in a 6.45-fold decrease in the odds favouring vulnerability to food insecurity. However, our study results contradict with Mota et al. [42], who found that in Damot Gale Woreda, Wolaita Zone, Southern Ethiopia, older household heads are more likely to experience food insecurity compared to younger ones, possibly due to decreased productivity and limited opportunities for off-farm income.

Within the model, factors such as gender (HHS), HHMS, HHRRs, HHCR, and HHOWN were identified as significant contributors to a family's vulnerability to food insecurity. Additionally, HHI, HHFE, and HHA played crucial roles in determining household food security. On the contrary, HHEdu, HHEMS, and HHFMZ were deemed insignificant in influencing household food security.

## 4. Conclusions

This study empirically examines food security challenges and identifies factors of food insecurity severity in Ethiopia's urban (Gondar) city. The study focussed on critical variables influencing household food insecurity such as female-headed households, unmarried household heads, and larger family sizes, which were particularly vulnerable to food insecurity. Additionally, economic factors such as HHI, HHFE, remittances, occupational status, and HHOWN played significant roles as determined with the help of inferential tests. The findings from the FGT index underscored the severity of the situation, with 53% of sampled households unable to meet minimum subsistence requirements in Gondar. The headcount ratio, food shortfall, and severity of food insecurity further emphasized the gravity of the issue. Moreover, a binary logistic regression model identified several significant predictor variables associated with household food security. Eight of the 11 chosen variables demonstrated significant associations, collectively exerting a substantial impact on predicting household food security. The model's explanatory power, evaluated through pseudo-*R*^2^, indicated that approximately 77.8% of the variance in food security could be explained by the combined independent variables, highlighting the model's effectiveness in capturing and elucidating the complexities of household food security dynamics. These findings emphasize the urgent need for targeted interventions and policy measures to address the multifaceted factors contributing to food insecurity in urban settings like Gondar City, Ethiopia, and similar contexts worldwide.

## 5. Policy Recommendations

Our study explored the following policy implications to combat the food insecurity upsurge menace, especially in developing nations:

➢ Our study recognizes the need for capacity-building initiatives to enhance the institutional capabilities of local governments, enabling them to implement effective policies such as introducing the public distribution system, which was initially established by the Indian government as a food security system.
➢ The design and implementation of targeted support programs aimed to enhance households food security by facilitating access to credit and savings institutions.
➢ Implement financial assistance and training initiatives to empower impoverished urban households, providing them with the skills and resources needed for sustainable improvement in food security.
➢ Advocate for strategic investments in infrastructure, such as transportation and storage facilities, to optimize the distribution and accessibility of food, directly impacting household food security.

## 6. Limitations and Future Research Direction

This study is limited by its focus on a sample of households from only three kebeles in Gondar City, which may not fully represent the broader diversity of income sources and poverty determinants across the entire region. The use of the FCS and the FGT index allowed us to assess the depth and severity of poverty within the selected kebeles, but the findings may not capture variations in poverty drivers that could exist in other kebeles with different economic activities and income sources. Consequently, the results may have limited generalizability beyond the studied areas. Further research should expand the scope to include additional kebeles or at a country level or at a group of country level, allowing for a more comprehensive analysis of poverty determinants. Future studies could also incorporate other factors influencing poverty, such as employment patterns, access to social services, and infrastructure, to provide a deeper understanding of the complexities of poverty in general.

## Figures and Tables

**Table 1:** Sample distribution.

**Sampled kebeles**	**Households** ^**a**^	**Sample size**	**Percentage**
Adebabay Evesus	16,537	99	28%
Abajalie	18,691	112	31%
Azezo Dimaza	24,282	145	41%
Total	59,512	357	100

^a^Source: Gondar City Administration, Ethiopia.

**Table 2:** Descriptions and expected signs of the variables.

**Variable name**	**Variable value**	**Description**	**Expected sign**
Dependent variable			
Household food security status	Dummy	1 if secure or 0 if insecure	
Independent variables			
Sex	Dummy	(1 = male, 0 = female)	Positive/negative
Age	Continuous	Measured in years	Positive
Marital status	Dummy	(0 = single, 1 = married)	Positive
Education level	Categorical	0 = Illiterate 1 = read and write 2 = secondary 3 = diploma and above	Positive
Size of family	Continuous	In numbers	Positive/negative
Income of household	Continuous	In birr	Positive
Occupational status	Categorical	0 = paid work 1 = daily labor 2 = merchant 3 = small scale 4 = pension	Positive
Ownership of house	Dummy	0 = no, 1 = yes	Positive/negative
Expenditure on food	Continuous	In birr	Positive
Remittances	Dummy	0 = no, 1 = yes	Positive
Access to credit	Dummy	0 = no, 1 = yes	Positive/negative

*Note:* Made by authors.

**Table 3:** Demographic characteristics.

**Demographic variables**		**Household's kilocalorie consumption/day**			**Chi-square test**
		**% food secure**	**% food insecure**	**Total**	
HHS	Male	43	21	(228) 64	91.30⁣^∗^
	Female	10	26	(129) 36	
	Total	53	47	(357) 100	

HHMS	Single	9	37	(164) 46	85.6⁣^∗∗^
	Married	37	17	(193) 54	
	Total	46	54	(357) 100	

HHFMZ	1–3	12	13	(89) 25	63.99⁣^∗∗^
	4–6	28	36	(228) 64	
	≥7	4	7	(39) 11	
	Total	44	56	(357) 100	

HHA	20–40	9	12	(75) 21	33.31⁣^∗^
	41–60	41	33	(264) 74	
	61–80	3	2	(18) 5	
	Total	53	47	(357) 100	

HHEdu	Illiterate	15	18	(118) 33	30.62⁣^∗^
	Read and write	13	10	(82) 23	
	Secondary	17	12	(103) 29	
	Diploma and above	8	7	(53) 15	
	Total	53	47	(357) 100	

*Note:* Source: authors' calculations.

⁣^∗∗^*p* < 0.001.

⁣^∗^*p* < 0.05.

**Table 4:** Socioeconomic characteristics.

**Socioeconomic characteristics**		**% food secure**	**% food insecure**	**Total**	**Chi-square test**
	**Ethiopian birr/month**				
Food expenditure	1500–2500	1	7	(28) 8	112.6⁣^∗^
	2501–3500	5	26	(111) 31	
	3501–4500	22	14	(128) 36	
	4501–5500	3	15	(64) 18	
	> 5500	7	0	(25) 7	
	Total	38	62	(357) 100	

Household income	< 2500	2	16	(64) 18	235.22⁣^∗∗^
	2501–4500	12	24	(128) 36	
	4501–6500	27	0	(96) 27	
	6501–8500	11	0	(39) 11	
	> 8500	8	0	(28) 8	
	Total	60	40	(357) 100	

Remittance received	Yes	30	5	(123) 35	99.07⁣^∗∗^
	No	25	40	(234) 65	
	Total	55	45	(357) 100	

Occupational status	Paid work	26	24	(178) 50	13.98⁣^∗∗^
	Daily labor	4	7	(39) 11	
	Merchant	6	13	(68) 19	
	Small scale	8	5	(46) 13	
	Pension	3	4	(25) 7	
	Total	47	53	(357) 100	

Housing ownership	Tenant	15	50	(232) 65	98.10⁣^∗^
	Home owner	34	11	(125) 35	
	Total	49	61	(357) 100	

Access to credit service	Yes	25	15	(143) 40	37.31⁣^∗∗^
	No	18	42	(214) 60	
	Total	43	57	(357) 100	

*Note:* Source: authors' calculations.

⁣^∗∗^*p* < 0.001.

⁣^∗^*p* < 0.05.

**Table 5:** Household food security status by FGT.

**FGT measures**	**Sum**	**FGT**	**% FGT**
(*P*_0_)	189	0.528	53
(*P*_1_)	54.085	0.1437	14
(*P*_2_)	19.34	0.067	5.4

*Note:* Source: authors' calculations.

**Table 6:** Binary logistic regression model.

**Predictor variables**		**Coefficients**	**SE**	**Wald**	**Sig.**	**Exp**	**95% CI for Exp**	
							**Lower**	**Upper**
Step 1	HHS	3.12	0.87	12.33	0.003⁣^∗^	14.56	3.56	62.34
	HHMS	2.45	0.754	11.67	0.002⁣^∗^	17.45	2.98	98.67
	HHRR	3.78	0.58	17.43	0.000⁣^∗^	75.23	15.43	356.87
	HHCR	3.12	0.678	14.45	0.000⁣^∗^	14.34	4.45	70.67
	HHOWN	2.67	0.745	20.56	0.000⁣^∗^	38.67	18.78	198.56
	Constant	−7.87	2.67	35.12	0.000	1.23		

Step 2	HHFE	5.67	2.34	18.76	0.000⁣^∗^	120.67	13.23	1243.05
	HHI	8.36	2.67	22.87	0.003⁣^∗^	198.45	47.34	2950.54
	HHA	2.34	0.78	8.43	0.0006⁣^∗^	6.45	3.143	25.76
	HHFMZ	−0.456	0.63	1.34	0.532	0.765	0.476	5.45
	HHEdu	−0.654	0.84	1.58	0.234	0.432	0.786	2.43
	HHEMS	1.37	0.46	1.67	0.358	2.45	0.943	2.68
	Constant	−15.45	2.98	28.45	0.004	0.003		

*Note:* Authors' calculation: *N* = 357; df = 1; Prob > chi^2^ = 2.41 (*p* < 0.01); pseudo-*R*^2^ = 0.7785. Hosmer and Lemeshow′s = 0.092.

⁣^∗^Significance at the level of 1%.

## Data Availability

Data used in the research would be made available on reasonable request.
